# Incidence and Predictors of Switching and Dose Change of Direct Oral Anticoagulants among Elderly Patients with Nonvalvular Atrial Fibrillation: A 5-Year Analysis of a Large Administrative Database

**DOI:** 10.3390/jcm12062379

**Published:** 2023-03-19

**Authors:** Leonardo De Luca, Melania Dovizio, Diego Sangiorgi, Valentina Perrone, Luca Degli Esposti

**Affiliations:** 1 Department of Cardio-Thoracic-Vascular Sciences, A.O. San Camillo-Forlanini, 00151 Rome, Italy; 2 Department of Cardio-Thoracic and Vascular Medicine and Surgery, U.O.C. Cardiologia, Azienda Ospedaliera San Camillo-Forlanini Circonvallazione Gianicolense, 87, 00152 Roma, Italy; 3 UniCamillus-Saint Camillus International, University of Health Sciences, 00131 Rome, Italy; 4 CliCon S.r.l. Società Benefit, Health, Economics & Outcomes Research, 40137 Bologna, Italy

**Keywords:** atrial fibrillation, direct oral anticoagulant agents, switching

## Abstract

In the last decade, novel oral anticoagulants (NOACs) have emerged as prominent therapeutic options in non-valvular atrial fibrillation (NVAF). We analysed the clinical burden and the switching rate between all available NOACs, and their dosage change over a period of 5 years in a representative population of patients with NVAF aged between 70 and 75 years. Methods and Results: This is a retrospective observational study on administrative databases, covering approximately 6.2 million health-assisted individuals by the Italian National Health System (around 11% of the entire Italian residents). Out of 4640 NVAF patients treated with NOACs and aged 70–75 years in 2017, 3772 (81.3%) patients were still in treatment with NOAC up to 2021 and among them, 3389 (73.0%) patients remained in treatment with the same NOAC during 2017–2021. In fact, 10.2% of patients switched NOAC type and 10.3% changed the dose of the same NOAC. Overall, after switching, the dabigatran and rivaroxaban groups lost, respectively, 13.5% and 2.8% of patients, while apixaban and edoxaban resulted in a relative percentage increase of 6.8% and 44.6% of patients, respectively. By a logistic regression analysis, the treatment with rivaroxaban, apixaban, and edoxaban (respect to dabigatran) was associated with a significant risk reduction of switch of 57%, 68%, and 44%, respectively. On the other hand, several features of high risk were associated with dose reduction. Conclusions. In our 5-year analysis of a large administrative database, a switching among NOACs or a change in NOAC dosages occurred in around 20% of elderly patients with NVAF. The type of NOAC was associated with a high switching rate, while several characteristics of high risk resulted as predictors of dose reduction of NOACs. Moreover, a worsening trend of clinical conditions occurred in patients maintaining the same NOAC treatment across 2017–2021.

## 1. Introduction

Atrial fibrillation (AF) is the most common sustained cardiac arrhythmia, and has affected approximately 7.6 million people over 65 years in European countries (in 2016), and this number will increase by 89%, to 14.4 million by 2060, with the prevalence expected to rise by 22%, from 7.8% to 9.5% [1]. Due to the progressive aging of the population, AF will become one of the major causes of stroke, heart failure, sudden death, and cardiovascular morbidity in the world with a significant impact on the economic burden for the National Health System (NHS) and society.

In the last decade, novel oral anticoagulants (NOACs) have emerged as prominent therapeutic alternatives to vitamin K antagonists (VKA), providing both clinicians and patients with more safe and effective treatment options in non-valvular atrial fibrillation (NVAF) [2,3,4,5,6]. After the introduction on the market of different NOACs, with various dosages to be used based on specific clinical characteristics, clinicians are faced with increasingly complex decisions relating to appropriate agents and dosing [7,8]. Accordingly, switching and dosage change could be an effective and appropriate strategy considering the clinical variations that often occur in patients with NVAF.

Changes in clinical characteristics and conditions occur more frequently in elderly patients, who are more prone to worsening indexes of frailty, renal dysfunction, the appearance of other comorbidities, and a greater risk of traumatic or haemorrhagic events [9,10].

To date, limited data are available on the analysis of switching and its predictors among NOACs in NVAF patients. Thus, a retrospective observational analysis was performed to evaluate, in a representative population of patients with NVAF aged between 70 and 75 years, the switching rate between all available NOACs and their dosage change over a period of 5 years. Moreover, the trend of clinical conditions and healthcare resource consumption in patients remaining on the same NOAC across 2017 to 2021 was evaluated.

## 2. Methods

### 2.1. Data Source

This is a retrospective observational study on data extracted from the administrative databases from a pool of Italian Healthcare Departments, geographically distributed across Italy, covering approximately 6.2 million health-assisted individuals by the Italian National Health System (INHS), corresponding to almost 11% of the entire Italian resident population. Data were extracted from the following databases: (i) demographic database, which consists of all patient demographic data, such as gender, age, death; (ii) pharmaceuticals database, which supplies information on medicinal products reimbursed by the INHS, such as the Anatomical Therapeutic Chemical (ATC) code, number of packages, number of units per package, unit cost per package, and prescription date; (iii) hospitalization database, which comprises all hospitalizations data for patients in analysis, such as the discharge diagnosis codes classified according to the International Classification of Diseases, Ninth Revision, Clinical Modification (ICD-9-CM), Diagnosis Related Group (DRG), and DRG-related charge (provided by the INHS), both as primary or secondary diagnosis; (iv) outpatient specialist services database, which incorporates all information about visits and diagnostic tests for patients under analysis (date and type of prescription, description of activity, and laboratory test or specialist visit charge); (v) payment exemption database, which contains data of the exemption codes that allow patients to avoid the contribution charge for services/treatments when specific diseases are diagnosed. For the current study, Italian Entities databases were selected by their geographical distribution (by north/centre/south of Italy), by data completeness, and by the high-quality linked datasets.

An anonymous univocal numeric code was assigned to each study individual to guarantee patients’ privacy, in full conformity with the European General Data Protection Regulation (GDPR) (2016/679). The patient code in each database permitted the electronic linkage among all databases. The results were produced as aggregated summaries and were never attributable to a single institution, department, doctor, individual, or individual prescribing behaviours. The analysis has been notified and approved by the local Ethics Committees of the Healthcare Departments involved in the study.

### 2.2. Study Design and Study Population

Among the population, all patients aged 70–75 years and with the first prescription of NOACs (i.e Dabigatran (ATC code: B01AE07, administered twice daily—standard dose: 150 mg; reduced dose: <150 mg, i.e., 110 mg), Rivaroxaban (ATC code: B01AF01, administered once daily—full dose: 20 mg; reduced dose: <20 mg, i.e., 15 mg), Apixaban (ATC code: B01AF02, administered twice daily—full dose: 5 mg; reduced dose: <5 mg, i.e., 2.5 mg), and Edoxaban (ATC code: B01AF03, administered once daily—full dose: 60 mg; reduced dose: <60 mg, i.e., 30 mg), from 2017 to 2021, and with a previous diagnosis of NVAF, were included. NVAF was identified by the hospitalization discharge diagnosis ICD-9 cm code 427.31 (at any levels) during the whole available period and before the index date (the first NOAC prescription during 2017).

### 2.3. Evaluation of Clinical Burden and Healthcare Resource Use Analysis

**Figure 1:**
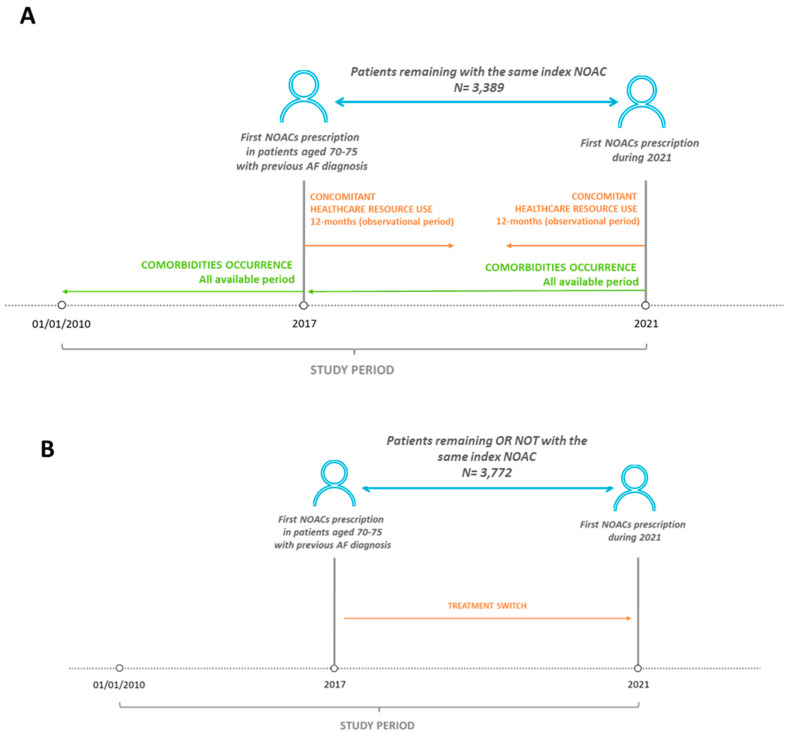
Schematic representation of study design. (**A**) For the analysis of comorbidities and healthcare resource consumption, patients prescribed with NOAC during 2017 and maintaining the same NOAC up to 2021 were included (N = 3389). (**B**) For the analysis of treatment switch, patients prescribed with NOAC during 2017 and remaining in treatment (with or without the same NOAC) up to 2021 were included (N = 3772).

To evaluate the trend of clinical variables and the healthcare resource consumption during 2017–2021, patients who continued the same index treatment from 2017 up to 2021 were identified (Figure 1A) and included in the analysis. Included patients were analysed during 2017 and 2021 and the index date corresponded to the first NOAC prescription during 2017 or 2021. The 2017–2021 time frame was selected to permit the inclusion of all NOAC molecules (during 2017) in the analysis and to follow NOAC-treated patients for the most prolonged period (up to 2021) based on database data availability.

The characterization period included all available periods (at least 12 months) before the index date. The follow-up (observational period) started from the index date up to 12 months after (for 2017 cohort) or before (for 2021 cohort) (Figure 1A).

The occurrence of co-morbidities was evaluated during all available periods before index date (before 2017 for the first cohort, and during 2017–2021 for the second cohort). These comorbidities were identified by applying diagnosis proxies based on hospitalization discharge diagnosis and specific medications (detailed in Supplementary Materials) [11,12]. Some of the variables included are the following: use of anti-diabetics, use of lipid-lowering agents, use of antihypertensives, tumours, heart failure, acute myocardial infarction (AMI), chronic kidney disease [13], trauma, rheumatoid arthritis, liver disease, use of osteoporosis medications, and chronic obstructive pulmonary disease (COPD). During the 12 months of observation, the consumptions of healthcare resources in terms of pharmaceutical prescriptions (evaluated for those drugs reimbursed by the Italian NHS), hospitalizations, and outpatient specialist service prescriptions, was evaluated. A detailed description of concomitant treatments (evaluated during NOAC therapy, as the 20 most frequent treatments based on the second ATC level) was reported.

### 2.4. Evaluation of Treatment Switch

Treatment switch was evaluated among NVAF patients who presented NOAC prescriptions during 2017 and were continuously followed up to 2021 (Figure 1B). In these patients, treatment switch was defined as the change of the index medication identified during 2017 with respect to the first NOAC prescribed during 2021; the switch rate was reported as the number and percentage of patients who changed the index NOAC from 2017 to 2021. Moreover, during the observational period of 12 months, daily dosage was estimated, and dose changes were calculated by considering the switch from a reduced to full, or a full to reduced dose across 2017 and 2021.

### 2.5. Statistical Analysis

Continuous variables were reported as mean and standard deviation (SD), while categorical variables were expressed as frequencies and percentages. The statistical significance was accepted for *p* values < 0.05. The standardized mean difference (SMD) was used to compare the different sub-cohorts for all variables considered; Cohen et al. suggested that SMD values above 0.2 be considered small, SMD values above 0.5 considered medium-sized, and SMD values above 0.8 considered large [14]. Moreover, a logistic regression model was used to analyse the probability of treatment switch and dose switch among treatment groups, adjusting for the following variables evaluated at baseline during 2017: age, sex, anti-diabetics use, lipid-lowering agents use, antihypertensives use, tumours, heart failure, AMI, chronic kidney disease, trauma, rheumatoid arthritis, liver disease, osteoporosis medication use, COPD, stroke, bleeding, and the index NOAC (dabigatran was considered as a reference in the analysis as the less recent medication available in Italy) over the other treated groups. The hazard ratio (HR) and 95% Confidence Interval (CI) was reported. According to “Opinion 05/2014 on Anonymization Techniques” drafted by the “European Commission Article 29 Working Party”, the analyses involving fewer than 3 patients were not reported, as they were potentially traceable to single individuals. Therefore, results referring to ≤3 patients were reported as NI (not issuable).

## 3. Results

**Figure 2:**
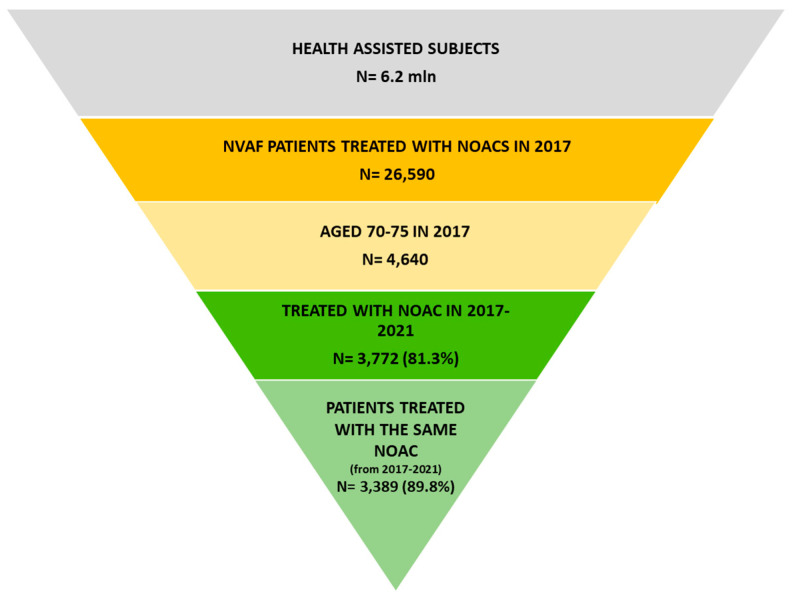
Flow chart of patients’ identification. Among 6.2 million health-assisted individuals, 26,590 patients with a diagnosis of NVAF and treated with NOAC during 2017 were identified. Among them, those aged 70–75 were selected (N = 4640). In total, 3772 patients prescribed with NOAC across 2017 and 2021 were identified, and among them 3389 patients, who in 2021 remained with the same NOAC started during 2017, were included.

From a sample population of 6.2 million health-assisted individuals, 26,590 NVAF patients treated with NOACs were identified (Figure 2). Among them, 4640 were aged 70–75 years in 2017, and 3772 (81.3%) patients were in treatment with NOAC between 2017 and 2021. Among them, 3389 (89.8%) patients remained in treatment with the same NOAC from 2017 up to 2021. Out of these 3389 patients, 885 (26%) were treated with dabigatran, 1259 (37%) with rivaroxaban, 1030 (31%) with apixaban, and 215 (6%) with edoxaban at the index date (2017). At inclusion, the mean age was 72.6 ± 1.8, 72.6 ± 1.7, 72.7, 72.6 ± 1.7, and 72.8, 72.6 ± 1.7 for dabigatran-, rivaroxaban-, apixaban, and edoxaban-treated patients, respectively. Among the different sub-groups, the occurrence of comorbidities during 2017 and 2021 was evaluated and reported in Table 1. Between 2017 and 2021, an increase in use of lipid-lowering medications (indicating an increase in hypercholesterolemia) (+15–23%), chronic kidney disease (+35–45%; and GFR < 60 +20–71%), use of osteoporosis medications (indicating osteoporosis occurrence) (+25–58%), trauma (+47–79%), COPD (+25–34%), and cancers (+15–35%) was found.

The clinical burden was also evaluated by analysing the most frequent concomitant drugs, evaluated during 12 months of the observation period (Supplementary Table S1). The healthcare resource consumptions in terms of drug prescriptions, hospitalizations, and specialist services (test/visits) are reported in Supplementary Table S2.

A focused analysis on NOAC pharmaco-utilization, in terms of treatment and dosage switch, was performed in all patients treated with NOAC during 2017–2021 (N = 3772) (Figure 2). As reported in Table 2, of 3772 NVAF patients who were NOAC-treated during 2017 up to 2021, 383 (10.2%) switched NOAC across 2017 and 2021. Overall, after switching, the dabigatran and rivaroxaban groups lost, respectively, 13.5% and 2.8% of patients, while apixaban and edoxaban resulted in a relative percentage increase of 6.8% and 44.6% of patients, respectively.

By a logistic regression analysis, the predictors of treatment switch were identified (Supplementary Table S3). The presence of previous use of antihypertensive medications was associated with a 32% reduction in risk of switching; the treatment with rivaroxaban, apixaban, and edoxaban (respect to dabigatran) was associated with a significant reduction in risk of switching of 57%, 68%, and 44%, respectively.

Among 3772 elderly patients with NVAF, a treatment dose change occurred in 10.3% of cases during the 5 years of observation (Table 3). By a logistic regression analysis, among patients who remained with the same index medication, the presence of trauma, previous use of anti-diabetic drugs, history of AMI, and heart failure were associated with an increased risk of dose change; apixaban (respect to dabigatran) was associated with a reduction in risk of dose change (Supplementary Table S4).

## 4. Discussion

The present analysis of administrative databases of a large cohort of elderly patients with NVAF showed that over 5 years, a worsening trend of clinical conditions which increased both thrombotic and haemorrhagic risk occurred. Nevertheless, a relatively low incidence of switching was observed in terms of type and dose of NOACs.

Oral anticoagulation therapy (OAC) has been demonstrated to significantly decrease the risk of stroke in patients with NVAF [2,3,4,5,6]. NOACs are associated with comparable efficacy to VKAs with better safety profiles [2,3,4,5,6]. Since their approval, the use of NOACs has been increasing, with a concomitant decline in warfarin utilization [15,16,17]. Notably, the availability of multiple NOACs created an opportunity to switch among them, potentially contributing to practice variation. Switching between NOACs is a prescriber’s choice, which may be related to several clinical-, patient-, or drug-related issues, while a change in dose of NOACs should be strictly related to patients’ characteristics in compliance with current recommendations and labelling/packaging inserts [18]. However, to date, several studies suggest inappropriate criteria for changing the type and dosing of NOACs [19,20] and no studies combining predictors of switching between NOAC and a reduction in their dosage have been published. In our analysis, a treatment with dabigatran resulted as the positive predictor of switching with respect to other NOACs, while a history of cardiovascular diseases, trauma, and a treatment with apixaban (versus dabigatran) were independently associated with a change in dosing during the period of observation, possibly to improve its label appropriateness.

The switching rate of NOACs ranges from 3 to 30% and varies depending on the timing of the authorisation of each NOAC, the duration of follow-up, study population, and the type of molecule [21,22]. As by the current analysis, switching rates are usually higher for dabigatran than for any other OAC, and it was usually the least preferred agent to switch to from another OAC in all studies published to date [18]. This finding has been confirmed even in our analysis and may be related to a poorer safety profile, twice-daily dosing and the imminent generication that could lead to doubts on the effectiveness of equivalent drugs placed on the market. In accordance with our data, in another analysis performed in Italy that enrolled NOAC-initiated patients between 2015 and 2017, the switching rate from the index NOAC to another NOAC within 24 months of initiation was 3.3%, with most of the switching arising from dabigatran-treated patients [23].

Advanced age, which can increase the risk of bleeding and stroke, particularly when other conditions, such as active cancer, subsist [24], has been usually associated with a lower likelihood of switching among NOACs [18,25,26,27], especially in Europe [28]. In our series of elderly patients with NVAF, a switching among NOAC occurred in approximately 10% during the 5 years of observation, with the highest percentage increase in favour of edoxaban, probably due to the fact that it is the most recently introduced molecule on the market. In addition, advances in knowledge on safety in the most fragile patients or the simplicity and handling of edoxaban may have increased its use [29,30]. To the best of our knowledge, this is the first analysis on switching that has included all four NOACs, and therefore supports even more the greater percentage who switch to edoxaban, although it must be considered that, even in our analysis, it has only recently been launched on the Italian market.

## 5. Study Limitations

Several limitations, inherent to the observational nature of the data, should be acknowledged. First, some unmeasured and residual confounding, including selective prescribing and other baseline characteristics, might persist despite the adjustment performed by logistic models. Unmeasurable baseline factors, such as biological age or severity of the co-morbidities, might affect both the choice of medications and the outcomes, leading to confounding results. Since the comorbidities analysed in the current manuscript were addressed based on any available data before inclusion (using a proxy of diagnosis, which includes disease-specific hospitalization and/or specific medications), there might be incomplete capture of these variables among patients. Moreover, patients included in the study had a hospital-confirmed diagnosis of NVAF, meaning that patients treated in the primary care setting—potentially with less severe general health status—were not captured. As in the vast majority of these observational studies, adjustment for covariates was carried out with covariates scored at baseline only, thus the impact of other variables was not evaluated for the analysis of switch predictors. Moreover, multiple switching/multiple dose change among NOACs during 5 years was not evaluated in the current analysis.

## 6. Conclusions

In our 5-year analysis of an administrative database accounting for more than 6 million health-assisted individuals, switching among NOACs or a change in NOAC dosages occurred in around 10% of elderly patients with NVAF. The type of NOAC was associated with a high switching rate, while several characteristics of high risk resulted as predictors of dose reduction of NOACs. Moreover, a worsening trend of clinical conditions which increased both thrombotic and haemorrhagic risk occurred in patients who maintained the same NOAC treatment across 2017–2021. Prospective studies are needed to assess the actual incidence of switching among NOACs and the appropriateness and predictors of this strategy in large and unselected populations of patients with NVAF.

## Figures and Tables

**Table 1:** Evaluation of comorbidities occurrence in patients remaining with the same NOAC across 2017 and 2021 (N = 3389).

	Dabigatran N = 885			Rivaroxaban N = 1259			Apixaban N = 1030			Edoxaban 215		
	2017	2021	Δ%	2017	2021	Δ%	2017	2021	Δ%	2017	2021	Δ%
Antihypertensives use (n, %)	839 (94.8)	865 (97.7)	3.1	1127 (89.5)	1228 (97.5)	9.0	942 (91.5)	1003 (97.4)	6.5	189 (87.9)	208 (96.7)	10.1
Lipid-lowering agents use (n, %)	454 (51.3)	522 (59.0)	15.0	610 (48.5)	737 (58.5)	20.8	491 (47.7)	596 (57.9)	21.4	104 (48.4)	128 (59.5)	23.1
Rheumatoid arthritis (n, %)	6 (0.7)	10 (1.1)	66.7	15 (1.2)	18 (1.4)	20.0	4 (0.4)	5 (0.5)	25.0	<4	<4	/
Chronic kidney disease (n, %)	159 (18.0)	229 (25.9)	44.0	249 (19.8)	358 (28.4)	43.8	238 (23.1)	345 (33.5)	45.0	46 (21.4)	62 (28.8)	34.8
GFR < 60 (n, %)	30 (3.4)	36 (4.1)	20.0	50 (4.0)	66 (5.2)	32.0	33 (3.2)	57 (5.5)	72.7	7 (3.3)	12 (5.6)	71.4
Osteoporosis medication use (n, %)	45 (5.1)	61 (6.9)	35.6	54 (4.3)	82 (6.5)	51.9	62 (6.0)	98 (9.5)	58.1	12 (5.6)	15 (7.0)	25.0
Trauma (n, %)	69 (7.8)	102 (11.5)	47.8	72 (5.7)	129 (10.2)	79.2	75 (7.3)	110 (10.7)	46.7	15 (7.0)	22 (10.2)	46.7
COPD (n, %)	248 (28.0)	333 (37.6)	34.3	353 (28.0)	447 (35.5)	26.6	303 (29.4)	399 (38.7)	31.7	57 (26.5)	71 (33.0)	24.6
Anti-diabetics use (n, %)	203 (22.9)	246 (27.8)	21.2	280 (22.2)	335 (26.6)	19.6	225 (21.8)	272 (26.4)	20.9	39 (18.1)	44 (20.5)	12.8
AMI (n, %)	211 (23.8)	252 (28.5)	19.4	255 (20.3)	301 (23.9)	18.0	212 (20.6)	252 (24.5)	18.9	53 (24.7)	60 (27.9)	13.2
Heart failure (n, %)	148 (16.7)	184 (20.8)	24.3	254 (20.2)	327 (26.0)	28.7	245 (23.8)	309 (30.0)	26.1	53 (24.7)	62 (28.8)	17.0
Cancer (n, %)	59 (6.7)	94 (10.6)	59.3	75 (6.0)	110 (8.7)	46.7	80 (7.8)	119 (11.6)	48.8	15 (7.0)	24 (11.2)	60.0
Liver disease (n, %)	26 (2.9)	35 (4.0)	34.6	48 (3.8)	60 (4.8)	25.0	39 (3.8)	47 (4.6)	20.5	13 (6.0)	15 (7.0)	15.4

**Table 2:** Switch of NOAC treatment across 2017–2021 among patients under NOAC (N = 3772).

Treatment Switch across 2017–2021	N	Dabigatran, 2021	Rivaroxaban, 2021	Apixaban, 2021	Edoxaban, 2021
Dabigatran, 2017	1062	-	47 (4.4)	76 (7.2)	54 (5.1)
Rivaroxaban, 2017	1373	21 (1.5)	-	53 (3.9)	40 (2.9)
Apixaban, 2017	1097	9 (0.8)	20 (1.8)	-	38 (3.5)
Edoxaban, 2017	240	4 (1.7)	8 (3.3)	13 (5.4)	-
TOTAL (2017–2021% variation)	3772	919 (−13.5%)	1334 (−2.8%)	1172 (+6.8%)	347 (+44.6%)

**Table 3:** Treatment switches between 2017 and 2021 among patients under NOAC (N = 3772), according to dosage.

Treatment Switch across 2017–2021	N	Dabigatran Reduced Dose, 2021	Dabigatran Standard Dose, 2021	Rivaroxaban Low Dose, 2021	Rivaroxaban Standard Dose, 2021	Apixaban Low Dose, 2021	Apixaban Standard Dose, 2021	Edoxaban Low Dose, 2021	Edoxaban Standard Dose, 2021
Dabigatran reduced dose, 2017	327	*-*	45 (13.8)	<4	7 (2.1)	10 (3.1)	15 (4.6)	8 (2.4)	7 (2.1)
Dabigatran standard dose, 2017	735	72 (9.8)	*-*	5 (0.7)	32 (4.4)	4 (0.5)	47 (6.4)	8 (1.1)	31 (4.2)
Rivaroxaban low dose, 2017	227	<4	0 (0.0)	*-*	42 (18.5)	6 (2.6)	10 (4.4)	7 (3.1)	0 (0.0)
Rivaroxaban standard dose, 2017	1146	7 (0.6)	13 (1.1)	111 (9.7)	*-*	9 (0.8)	28 (2.4)	12 (1.0)	21 (1.8)
Apixaban low dose, 2017	112	0 (0.0)	0 (0.0)	<4	<4	*-*	39 (34.8)	5 (4.5)	<4
Apixaban standard dose, 2017	985	<4	6 (0.6)	8 (0.8)	10 (1.0)	47 (4.8)	*-*	9 (0.9)	23 (2.3)
Edoxaban low dose, 2017	56	<4	0 (0.0)	0 (0.0)	0 (0.0)	<4	<4	*-*	8 (14.3)
Edoxaban standard dose, 2017	184	0 (0.0)	<4	4 (2.2)	4 (2.2)	<4	8 (4.3)	24 (13.0)	*-*
total (2017–2021 % switch variation)	3772	316 (−3.4)	603 (−18.0)	293 (29.1)	1041 (−9.2)	143 (27.7)	1029 (4.5)	116 (107.1)	231 (25.5)

## Data Availability

Data are available upon request at Clicon.

## Supplementary Materials

The following supporting information can be downloaded at https://www.mdpi.com/article/10.3390/jcm12062379/s1.
