# Supplementary material for: Incidence and Predictors of Switching and Dose Change of Direct Oral Anticoagulants among Elderly Patients with Nonvalvular Atrial Fibrillation: A 5-Year Analysis of a Large Administrative Database
Source: J Clin Med. 2023 Mar 19;12(6):2379. doi: 10.3390/jcm12062379 (PMC10056372; doi:10.3390/jcm12062379)
Supplement: Supplementary file 1 [file jcm-12-02379-s001.zip › jcm-2272089-supplementary.pdf]

Supplementary Materials

List of comorbidities. ***Anti-diabetics***: at least one prescription of drugs with ATC code A10; ***Lipid-lowering agents***: at least one prescription of drugs with ATC C10; ***Antihypertensives***: at least one prescription of drugs with ATC codes C02, C03, C07, C08, C09; ***Tumors***: at least one hospitalization with discharge diagnosis codes ICD-9-CM 140-209, or at least one prescription of drugs with ATC code L01, or with an exemption code 048; ***COPD***: at least one prescription of drugs with ATC code R03; ***Stroke***: at least one hospitalization with discharge diagnosis codes ICD-9-CM 430-438; ***Heart failure***: at least one hospitalization with discharge diagnosis code ICD-9-CM 428; ***Acute Myocardial Infarction*** (AMI): at least one hospitalization with discharge diagnosis codes ICD-9-CM 410-414; ***chronic kidney disease***: at least one hospitalization with discharge diagnosis code ICD-9-CM 585, or at least one prescription of drugs with ATC codes A12AA, A02AH B03XA, V03AE02, V03AE03, V03AE04, A11CC03, A11CC04, or when available with GFR value<60 from laboratory test database; ***Trauma***: at least one hospitalization with discharge diagnosis codes ICD-9-CM codes 800-959; ***Rheumatoid arthritis***: at least one hospitalization with discharge diagnosis code ICD-9-CM 714, or an exemption code of 006; ***Liver disease***: at least one hospitalization with discharge diagnosis codes ICD-9-CM 570-573, 070; ***Osteoporosis medication***: at least one prescription of drugs with ATC codes M05BA, M05BB, M05BX, H05AA, H05BA, G03XC; ***Obesity***: at least one hospitalization with discharge diagnosis codes ICD-9-CM 278.0, 649.1, 793.9, V85.3, V85.4; ***Bleedings***: at least one hospitalization with discharge diagnosis codes ICD-9-CM 531.0x, 531.2x, 531.4x, 531.6x, 532.0x, 532.2x, 532.4x, 532.6x, 533.0x, 533.2x, 533.4x, 533.6x, 534.0x, 534.2x, 534.4x, 534.6x, 535.01, 535.11, 535.21, 535.31, 535.41, 535.51, 535.61, 537.83, 456.0, 456.20, 530.7,530.82, 578.0, 455.2, 455.5, 455.8, 562.02, 562.03, 562.12, 562.13, 568.81, 569.3, 569.85, 578.1, 578.9, 593.81, 599.7, 623.8, 626.2, 626.6, 423.0, 459.0, 719.1x, 784.7, 784.8, 786.3, 852.0, 852.2, 852.4, 853.0, 423.0, 568.81, 719.1x

Supplementary Table S1. Concomitant medications in patients remaining with the same NOAC between 2017-2021 (N= 3,389) , based on the second level of ATC

	Dabigatran, 2017	Dabigatran, 2021	Δ	Rivaroxaban, 2017	Rivaroxaban, 2021	Δ	Apixaban, 2017	Apixaban, 2021	Δ	Edoxaban, 2017	Edoxaban, 2021	Δ
Agents acting on the renin-angiotensin system	688 (77.1)	680 (76.2)	**-1.2**	959 (75.8)	935 (73.9)	**-2.5**	772 (74.4)	741 (71.5)	**-4.0**	167 (75.2)	157 (70.7)	**-6.0**
Beta blocking agents	621 (69.6)	633 (71.0)	**1.9**	894 (70.6)	908 (71.7)	**1.6**	716 (69.0)	739 (71.3)	**3.2**	148 (66.7)	146 (65.8)	**-1.4**
Drugs for acid related disorders	628 (70.4)	596 (66.8)	**-5.1**	826 (65.2)	747 (59.0)	**-9.6**	742 (71.6)	669 (64.5)	**-9.8**	149 (67.1)	127 (57.2)	**-14.8**
Antibacterials for systemic use	544 (61.0)	502 (56.3)	**-7.7**	749 (59.2)	700 (55.3)	**-6.5**	654 (63.1)	578 (55.7)	**-11.6**	125 (56.3)	119 (53.6)	**-4.8**
Lipid modifying agents	460 (51.6)	472 (52.9)	**2.6**	639 (50.5)	680 (53.7)	**6.4**	514 (49.6)	555 (53.5)	**8.0**	110 (49.5)	113 (50.9)	**2.7**
Cardiac therapy	484 (54.3)	457 (51.2)	**-5.6**	657 (51.9)	588 (46.4)	**-10.5**	578 (55.7)	512 (49.4)	**-11.4**	120 (54.1)	111 (50.0)	**-7.5**
Diuretics	395 (44.3)	408 (45.7)	**3.3**	609 (48.1)	649 (51.3)	**6.6**	514 (49.6)	553 (53.3)	**7.6**	99 (44.6)	107 (48.2)	**8.1**
Antiinflammatory	314 (35.2)	287 (32.2)	**-8.6**	410 (32.4)	389 (30.7)	**-5.1**	364 (35.1)	313 (30.2)	**-14.0**	77 (34.7)	63 (28.4)	**-18.2**
Vitamins	215 (24.1)	249 (27.9)	**15.8**	324 (25.6)	347 (27.4)	**7.1**	284 (27.4)	312 (30.1)	**9.9**	51 (23.0)	68 (30.6)	**33.3**
Calcium channel blockers	196 (22.0)	202 (22.6)	**3.1**	332 (26.2)	319 (25.2)	**-3.9**	278 (26.8)	268 (25.8)	**-3.6**	58 (26.1)	60 (27.0)	**3.4**
Drugs for obstructive airway diseases	236 (26.5)	221 (24.8)	**-6.4**	315 (24.9)	269 (21.2)	**-14.6**	301 (29.0)	262 (25.3)	**-13.0**	45 (20.3)	41 (18.5)	**-8.9**
Drugs used in diabetes	213 (23.9)	238 (26.7)	**11.7**	306 (24.2)	323 (25.5)	**5.6**	237 (22.9)	259 (25.0)	**9.3**	38 (17.1)	42 (18.9)	**10.5**
Antigout preparations	157 (17.6)	193 (21.6)	**22.9**	239 (18.9)	296 (23.4)	**23.8**	199 (19.2)	259 (25.0)	**30.2**	50 (22.5)	52 (23.4)	**4.0**
Antithrombotic agents	216 (24.2)	173 (19.4)	**-19.9**	276 (21.8)	222 (17.5)	**-19.6**	226 (21.8)	195 (18.8)	**-13.7**	60 (27.0)	36 (16.2)	**-40.0**
Corticosteroids for systemic use	138 (15.5)	156 (17.5)	**13.0**	204 (16.1)	194 (15.3)	**-4.9**	212 (20.4)	194 (18.7)	**-8.5**	39 (17.6)	39 (17.6)	**0.0**
Urologicals	159 (17.8)	176 (19.7)	**10.7**	195 (15.4)	225 (17.8)	**15.4**	154 (14.9)	180 (17.4)	**16.9**	45 (20.3)	41 (18.5)	**-8.9**
Analgesics	123 (13.8)	153 (17.2)	**24.4**	196 (15.5)	206 (16.3)	**5.1**	175 (16.9)	179 (17.3)	**2.3**	27 (12.2)	41 (18.5)	**51.9**
Psychoanaleptics	119 (13.3)	130 (14.6)	**9.2**	180 (14.2)	195 (15.4)	**8.3**	168 (16.2)	185 (17.8)	**10.1**	25 (11.3)	31 (14.0)	**24.0**
Antidiarrheals and intestinal agents	101 (11.3)	92 (10.3)	**-8.9**	147 (11.6)	140 (11.1)	**-4.8**	134 (12.9)	131 (12.6)	**-2.2**	34 (15.3)	30 (13.5)	**-11.8**
Antianemic preparations	80 (9.0)	115 (12.9)	**43.8**	117 (9.2)	150 (11.8)	**28.2**	110 (10.6)	126 (12.2)	**14.5**	25 (11.3)	22 (9.9)	**-12.0**
**At least one ATC, DOAC excluded**	**885 (100.0)**	**885(100.0)**	**0.0**	**1256 (99.8)**	**1255 (99.7)**	**0.0**	**1026 (99.6)**	**1028 (99.8)**	**0.0**	**214 (99.5)**	**214 (99.5)**	**0.0**

Supplementary Table S2. Healthcare resource consumption during 1 year of observation in patients remaining with the same NOAC between 2017-2021 (N= 3,389)

	**Dabigatran**		**Rivaroxaban**		**Apixaban**		**Edoxaban**	
	**2017**	**2021**	**2017**	**2021**	**2017**	**2021**	**2017**	**2021**
**Drugs, different ATC (mean, SD)**	10.9 (4.7)	11.0 (5.0)	10.8 (5.2)	10.7 (5.2)	11.3 (5.4)	11.2 (5.6)	11.0 (4.6)	10.6 (4.9)
**Hospitalizations (mean, SD)**	0.4 (0.8)	0.3 (0.7)	0.4 (0.8)	0.3 (0.6)	0.4 (0.8)	0.3 (0.7)	0.6 (1.1)	0.3 (0.7)
**Tests/visits (mean, SD)**	7.3 (7.4)	6.6 (6.4)	7.3 (6.6)	6.6 (5.6)	7.7 (6.7)	7.1 (6.5)	8.7 (6.4)	7.7 (7.0)

Supplementary Table S3. Logistic regression model for predictors of treatment switch in all patients treated with NOAC between 2017-2021 (N= 3,772)

	**OR**	**95% CI**		**P value**
Male	0.846	0.677	1.057	0.140
Age	0.997	0.937	1.060	0.917
Antihypertensives use	0.676	0.470	0.972	0.035
Lipid-lowering agents use	0.968	0.765	1.225	0.787
Rheumatoid arthritis	2.160	0.916	5.094	0.079
Chronic Kidney disease	1.072	0.819	1.404	0.613
Osteoporosis medication use	0.941	0.573	1.545	0.810
Trauma	0.878	0.563	1.370	0.567
COPD	1.145	0.903	1.451	0.264
Anti-diabetics use	1.087	0.832	1.419	0.541
AMI	0.906	0.680	1.206	0.498
Heart failure	1.062	0.807	1.396	0.669
Cancer	1.003	0.649	1.551	0.988
Liver disease	0.911	0.493	1.684	0.767
Bleeding	0.987	0.697	1.399	0.942
Stroke	0.862	0.638	1.166	0.337
Dabigatran	*REF.*			
Rivaroxaban	0.432	0.336	0.557	<0.001
Apixaban	0.315	0.233	0.424	<0.001
Edoxaban	0.557	0.356	0.872	0.011

Supplementary Table S4. Logistic regression model for predictors of dosage switch in all patients treated with NOAC between 2017-2021 (N= 3,772)

	**OR**	**95% CI**		**P**
Male	1.011	0.808	1.267	0.921
Age	1.036	0.973	1.102	0.271
Antihypertensives use	0.903	0.606	1.346	0.617
Lipid-lowering agents use	0.864	0.683	1.093	0.223
Rheumatoid arthritis	1.656	0.651	4.209	0.289
Chronic Kidney disease	1.164	0.899	1.507	0.249
Osteoporosis medication use	1.136	0.702	1.839	0.602
**Trauma**	**1.752**	**1.223**	**2.510**	**0.002**
COPD	1.115	0.882	1.410	0.363
Anti-diabetics use	**1.485**	**1.160**	**1.902**	**0.002**
**AMI**	**1.290**	**0.991**	**1.680**	**0.058**
**Heart failure**	**1.293**	**1.001**	**1.671**	**0.049**
Cancer	1.149	0.764	1.729	0.505
Liver disease	1.338	0.814	2.199	0.251
Bleeding	1.098	0.797	1.512	0.569
Stroke	1.193	0.908	1.568	0.206
Dabigatran	*REF.*			
Rivaroxaban	0.903	0.695	1.173	0.445
**Apixaban**	**0.572**	**0.424**	**0.771**	**<0.001**
Edoxaban	1.102	0.717	1.693	0.658
